# Beyond the Vasculature: The Emerging Role of Systemic Metabolism and Immunometabolism in Pulmonary Arterial Hypertension

**DOI:** 10.3390/ijms27062571

**Published:** 2026-03-11

**Authors:** Xin Chen, Xuezhu Wang, Raobin Xu, Shuang Gao, Jieru Han

**Affiliations:** 1 First Clinical Medical College, Heilongjiang University of Chinese Medicine, Harbin 150040, China; radinen@outlook.com (X.C.); wxzdyx2006@163.com (X.W.); 17209898832@163.com (R.X.); 2 School of Basic Medical Sciences, Heilongjiang University of Chinese Medicine, Harbin 150040, China; 13111547745@163.com

**Keywords:** pulmonary arterial hypertension, metabolic reprogramming, immunometabolism, obesity paradox, insulin resistance, organ crosstalk, therapeutic strategies

## Abstract

Pulmonary arterial hypertension (PAH) has traditionally been viewed as a vasculocentric disorder, with current therapies failing to reverse vascular remodeling or address pervasive systemic metabolic abnormalities. This review synthesizes emerging evidence to propose a paradigm shift, conceptualizing PAH as a systemic metabolic–immunological network disease. It examines how metabolic dysfunction in peripheral organs (adipose tissue, liver, skeletal muscle) and immunometabolic reprogramming of immune cells (e.g., macrophages, lymphocytes) synergistically drive pathology. These components engage in dynamic crosstalk via circulating mediators (metabolites, adipokines, cytokines), creating a self-amplifying loop that fuel pulmonary vascular inflammation and remodeling. Key mechanisms explored include adipose tissue endocrine dysfunction (contributing to the obesity paradox), hepatic insulin resistance and bile acid signaling, skeletal muscle energy crisis and wasting, and the pivotal roles of macrophage glycolytic polarization and T-cell subset imbalance. Insulin resistance/hyperglycemia emerges as a central hub linking metabolic and immune dysregulation. The review concludes that future therapeutic strategies must move beyond vasodilation to target this systemic network, discussing the potential of repurposed metabolic agents, direct immunometabolic modulators, and integrated lifestyle interventions to disrupt disease progression.

## 1. Introduction: Why Look Beyond the Vasculature?

PAH has traditionally been viewed as a disease centered on the pulmonary circulation. Its classic pathological mechanisms focus on pulmonary vascular remodeling, involving the abnormal proliferation of pulmonary arterial smooth muscle cells (PASMCs), endothelial dysfunction, and perivascular fibrosis, which lead to progressively increased pulmonary vascular resistance (PVR) and ultimately right ventricular failure [1,2]. While current vasodilatory and anti-proliferative therapies can alleviate symptoms, they fail to reverse vascular remodeling or address the widespread systemic metabolic abnormalities, indicating fundamental limitations in the traditional vascular-centric paradigm [3].

A series of clinical observations challenge the theory of isolated vascular pathology. Most notably, the “obesity paradox”—where some obese PAH patients may exhibit better survival rates—contradicts the conventional view that obesity exacerbates cardiovascular risk [4,5]. Furthermore, the prevalence of metabolic comorbidities such as insulin resistance/diabetes and muscle wasting is exceptionally high in PAH patients [6]. These systemic manifestations cannot be explained by localized pulmonary vascular pathology alone. Concurrently, PAH patients exhibit persistently elevated levels of circulating pro-inflammatory cytokines (e.g., IL-6, TNF-α), providing clear evidence of a systemic inflammatory process [7,8]. The paradoxes and evidence collectively point to PAH being far more than a localized disease.

Consequently, there is an urgent need for a new integrative paradigm to understand PAH. The core argument of this review is that metabolic dysfunction in peripheral organs (e.g., adipose tissue, liver, skeletal muscle) and the metabolic reprogramming of the immune system (e.g., macrophages, T lymphocytes) are key synergistic factors driving and exacerbating PAH. They engage in dynamic “crosstalk” with the pulmonary vasculature by releasing circulating messengers such as metabolites, adipokines, and cytokines, together constituting a complex systemic metabolic–immune–vascular network disorder. Moving beyond a purely vascular perspective to systematically examine this network will provide critical insights for deepening our understanding of PAH pathogenesis, explaining clinical paradoxes, and developing novel therapeutic strategies (Table 1).

## 2. Foundational Concepts: Core Principles of Metabolism and Immunometabolism

Under physiological conditions, cells possess “metabolic flexibility,” the ability to efficiently switch between different metabolic pathways (e.g., glucose/fatty acid oxidative phosphorylation, glycolysis) in response to energy demands, substrate availability, and signaling cues. This flexibility is fundamental to maintaining tissue functional homeostasis. For example, quiescent cells primarily rely on efficient mitochondrial oxidative phosphorylation for ATP production, while they can transiently upregulate glycolysis under conditions of rapid proliferation or hypoxia to provide intermediates and energy. This adaptive metabolic program is precisely regulated by key metabolic sensors and transcription factors such as AMPK and HIF [21].

Focusing on the immune system, the concept of “immunometabolism” has given rise, revealing a fundamental coupling between the functional state of immune cells and their metabolic programs. A classic paradigm is that pro-inflammatory immune cells (e.g., activated M1 macrophages, effector T cells) typically exhibit a fermentative metabolic profile, characterized by enhanced glycolysis and glutaminolysis akin to the “Warburg effect,” to rapidly generate ATP and biosynthetic precursors supporting their proliferation, migration, and burst secretion of inflammatory cytokines (e.g., IL-1β, TNF-α). Conversely, cells with anti-inflammatory or immunoregulatory functions (e.g., reparative M2 macrophages, regulatory T cells) rely more heavily on oxidative phosphorylation to sustain their long-term survival and homeostatic functions [22,23]. Thus, metabolic state is not merely a consequence but a key determinant shaping immune cell phenotypes.

**Figure 1:**
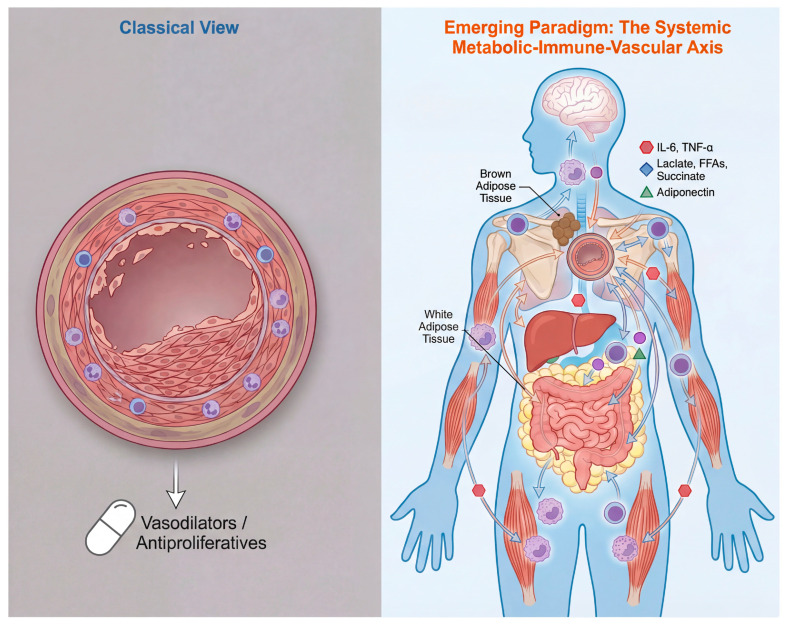
Paradigm shift in PAH: from a vasculocentric disease to a systemic metabolic–immune–vascular disorder. (**Left**) The classical view focuses on localized pulmonary vascular pathology, including endothelial dysfunction, smooth muscle cell proliferation, and inflammatory infiltration, leading to vascular remodeling and right heart failure. Therapies primarily target vasoconstriction and proliferation within the vessel. (**Right**) The emerging paradigm conceptualizes PAH as a systemic disorder. Metabolic and inflammatory dysregulation in key peripheral organs (adipose tissue, liver, skeletal muscle) and within circulating/resident immune cells generates a milieu of dysregulated signaling molecules (metabolites, adipokines, cytokines). These factors circulate and converge on the pulmonary vasculature, driving its pathology. This model integrates clinical phenomena like the “obesity paradox” and insulin resistance.

Applying this framework to PAH, a core pathological feature is the widespread loss of metabolic flexibility and the fixation into persistent, pathological metabolic programs. Both pulmonary vascular cells (including pulmonary arterial endothelial and smooth muscle cells) and infiltrating immune cells (e.g., macrophages, T cells) exhibit significant metabolic reprogramming: for instance, they adopt a metabolism dominated by glycolysis with impaired mitochondrial function, even in oxygen-replete environments [21,24]. This “metabolic lock” confers proliferative advantages and resistance to apoptosis, directly driving vascular remodeling. Crucially, the dysregulated systemic metabolic environment in PAH patients (e.g., insulin resistance, abnormal lipid metabolism) provides the “fuel” and signals for this pathological metabolic reprogramming of local cells, creating a vicious cycle that exacerbates disease progression [6,25]. Thus, these concepts provide the foundation for understanding how PAH evolves into a systemic metabolic–immune network disorder (Figure 1).

## 3. Peripheral Organ Metabolic Dysfunction: How Remote Organs Influence the Pulmonary Vasculature

### 3.1. Adipose Tissue: The Dysfunctional Endocrine Organ

#### 3.1.1. Altered Secretome in Dysfunctional Fat

The transition of adipose tissue from a metabolic regulator to a source of pathogenic signals is driven by profound alterations in its secretome, a process initiated by adipocyte hypertrophy and tissue hypoxia. In obesity, enlarged adipocytes outgrow their blood supply, creating local hypoxic zones that stabilize hypoxia-inducible factor-1α (HIF-1α). HIF-1α, in turn, upregulates the expression of pro-inflammatory genes (e.g., IL6, TNF) and promotes fibrosis, fundamentally reshaping the tissue microenvironment [26]. This hypoxic, stressed milieu triggers the recruitment and activation of immune cells, most notably macrophages. These infiltrating macrophages undergo metabolic reprogramming, shifting towards glycolytic metabolism which fuels a pro-inflammatory (M1) phenotype [27]. This creates a vicious cycle: activated M1 macrophages secrete cytokines like TNF-α and IL-6, which further impair adipocyte function, promote insulin resistance, and amplify the production of deleterious adipokines such as leptin and resistin, while suppressing beneficial ones like adiponectin [26,27]. Consequently, the adipose tissue secretome becomes dominated by factors that promote systemic inflammation, oxidative stress, and endothelial dysfunction—key drivers of remote organ damage, including pulmonary vascular remodeling [28,29].

#### 3.1.2. “Harmful” vs. “Protective” Adipokines: Leptin and Adiponectin

The balance between “harmful” and “protective” adipokines critically influences PAH pathogenesis. Leptin, elevated in obesity and PAH, binds to its receptor (ObR) on PASMCs, activating JAK2/STAT3, MAPK, and PI3K/Akt pathways, thereby promoting proliferation, migration, and apoptosis resistance [16]. It also skews macrophages toward a pro-inflammatory phenotype and impairs Treg suppressive function [16,30]. Conversely, adiponectin signals via AdipoR1/R2 to activate AMPK and PPARα, enhancing endothelial nitric oxide (NO) production, suppressing PASMC proliferation via mTOR inhibition, and promoting anti-inflammatory macrophage polarization (M2) [31]. The resulting high leptin/adiponectin ratio thus creates a permissive environment for pulmonary vascular disease [32].

#### 3.1.3. Potential Explanations for the Obesity Paradox

The “obesity paradox” in PAH suggests that the relationship between adipose tissue and outcomes is nonlinear and multifaceted, potentially explained by several interrelated mechanisms:

- Adipose Tissue Distribution and Quality:

The paradox may hinge not on total fat mass, but on its anatomical distribution and metabolic health. Subcutaneous adipose tissue (SAT), particularly the gluteofemoral depot, is more metabolically benign and may retain better adiponectin-secreting capacity compared to visceral adipose tissue (VAT), which is highly inflammatory [15,16]. Obese PAH patients with predominant SAT might be shielded by higher levels of protective adipokines [14]. Furthermore, the functionality of adipose tissue, including its angiogenic capacity and mitochondrial health, varies individually and may influence systemic metabolite profiles [33,34].

- Estrogen Metabolism and Signaling:

Adipose tissue is a major site for aromatase-mediated conversion of androgens to estrogens [35]. In obesity, this metabolism can be dysregulated, leading to increased production of pathogenic estrogen metabolites like 16α-hydroxyestrone (16αOHE1), which promotes oxidative stress and remodeling [36]. However, in some contexts, particularly in premenopausal women, adequate levels of “good” estrogens (e.g., 17β-estradiol) may provide vascular protection through antioxidant, anti-proliferative, and NO-promoting effects [37]. The paradox may thus reflect a complex interplay where obesity in some individuals (e.g., premenopausal women with functional SAT) does not fully abrogate these protective hormonal pathways [16,36,38,39].

- Metabolic Reserve and Adipokine Isoforms:

Obesity provides a larger energy reservoir, which might be advantageous in a catabolic state like advanced PAH. Additionally, adiponectin circulates in various multimeric forms (low-, middle-, and high-molecular-weight) [40,41]. The high-molecular-weight (HMW) form is considered the most biologically active for metabolic benefits. Some obese individuals, potentially those with more metabolically healthy phenotypes, may maintain favorable HMW adiponectin levels, conferring cardioprotective effects that mitigate the risks of pulmonary vascular disease [31].

These mechanisms are not mutually exclusive and likely coexist, underscoring that the net effect of adipose tissue on PAH is determined by a delicate equilibrium between its pathogenic secretory profile and its residual capacity for beneficial endocrine and metabolic functions.

### 3.2. The Liver: Dysregulation of the Metabolic Integrator

The liver serves as a central metabolic integrator in PAH, where its dysregulation extends beyond classical detoxification and storage functions to actively influence systemic inflammation, vascular tone, and right ventricular (RV) performance. This dysregulation manifests through disrupted insulin signaling and aberrant bile acid metabolism, both of which contribute to the progression of PAH via hepatic secretion of inflammatory mediators and vasoactive substances.

#### 3.2.1. Insulin Resistance and Hepatic Inflammatory Signaling

Insulin resistance in the liver is a key metabolic disturbance in PAH that promotes systemic inflammation and pulmonary vascular remodeling. Hepatic insulin resistance leads to increased secretion of pro-inflammatory cytokines such as interleukin-6 (IL-6) and tumor necrosis factor-alpha (TNF-α), which exacerbate endothelial dysfunction and smooth muscle cell proliferation in the pulmonary vasculature. This inflammatory milieu is further amplified by the liver’s role as a primary source of acute-phase proteins and chemokines under conditions of metabolic stress.

A 2023 study of 96 Hispanic PAH patients demonstrated that insulin resistance and metabolic syndrome are closely linked to liver fibrosis severity. Patients with advanced fibrosis (F3–F4) had a significantly higher prevalence of diabetes (51.9% vs. 21.1%, *p* = 0.001) and metabolic syndrome risk factors, underscoring the liver’s role in integrating metabolic and inflammatory pathways in PAH [11]. Additionally, hepatic steatosis and non-alcoholic fatty liver disease (NAFLD) are common in PAH patients, contributing to systemic insulin resistance and increased release of liver-derived inflammatory mediators. These factors correlate with worse clinical outcomes, including reduced exercise capacity and elevated N-terminal pro-B-type Natriuretic Peptide (NT-proBNP) levels, reflecting more severe right heart dysfunction [11,42].

The liver also modulates pulmonary vascular tone through insulin-like growth factors and adipokines, which are dysregulated in insulin-resistant states. For instance, hepatocyte-derived macrophage migration inhibitory factor (MIF) is elevated in portopulmonary hypertension (PoPH) patients and correlates with pulmonary vascular resistance (PVR, r = 0.58, *p* = 0.006) [43]. MIF activates Nuclear Factor kappa-light-chain-enhancer of activated B cells (NF-κB) signaling in pulmonary artery smooth muscle cells (PASMCs), promoting proliferation and resistance to apoptosis—a hallmark of PAH pathogenesis [44]. Furthermore, peroxisome proliferator-activated receptor γ (PPARγ) agonists such as pioglitazone, which improve insulin sensitivity, have been shown to reduce pulmonary vascular remodeling in rodent models of PAH [44]. These preclinical findings highlight the therapeutic potential of targeting the hepatic metabolic–inflammatory axis, although clinical validation in PAH patients is still needed.

Mitochondrial dysfunction in the liver, often associated with insulin resistance, also exacerbates PAH through impaired energy metabolism and increased oxidative stress [45,46]. For example, disrupted glutaminolysis—a metabolic pathway supported by hepatic glutamine synthesis—is linked to Bone Morphogenetic Protein Receptor Type 2 (BMPR2) mutations in heritable PAH, leading to increased reactive oxygen species (ROS) production and vascular remodeling [24,47]. Thus, hepatic insulin resistance not only fuels systemic inflammation but also directly engages pulmonary vascular cells through soluble mediators and metabolic reprogramming, positioning the liver as a critical orchestrator of metabolic–inflammatory crosstalk in PAH [25,48].

#### 3.2.2. Bile Acid Metabolism as a Signaling Pathway

Bile acids, synthesized in the liver, are increasingly recognized as important signaling molecules that influence systemic metabolism, inflammation, and vascular function through receptors such as the farnesoid X receptor (FXR) and Takeda G protein-coupled receptor 5 (TGR5). In PAH, bile acid metabolism is frequently disrupted due to cholestasis secondary to congestive hepatopathy or portosystemic shunting, leading to altered bile acid composition and signaling [25,49,50].

Cholestasis, characterized by elevated serum bile acids, γ-glutamyltransferase (GGT), and alkaline phosphatase (ALP), is common in PAH patients, particularly those with right heart failure. A 2020 study of 407 idiopathic PAH (iPAH) patients in China reported that 42.3% had elevated GGT and 38.1% had elevated ALP at baseline [19]. These cholestatic markers were independent predictors of mortality, with hyperbilirubinemia carrying a hazard ratio (HR) of 4.29 (95% CI 1.21–15.27, *p* = 0.02) [19]. The accumulation of bile acids in systemic circulation can activate FXR and TGR5 on endothelial cells and immune cells, modulating NO production, oxidative stress, and inflammatory responses [51].

Through FXR activation, bile acids regulate lipid and glucose homeostasis, while TGR5 signaling promotes anti-inflammatory and vasodilatory effects via increased cyclic AMP (cAMP) production [51,52]. Dysregulated bile acid signaling in PAH may therefore contribute to endothelial dysfunction and impaired vasodilation. Furthermore, in portopulmonary hypertension, portosystemic shunts bypass hepatic clearance, allowing gut-derived bile acids and bacterial products to enter the systemic circulation and exacerbate pulmonary vascular inflammation [53,54]. A 2023 study of PoPH patients found that plasma free fatty acids (FFAs) were 40% higher than in iPAH patients (*p* < 0.001), and FFAs correlated with PVR (r = 0.42, *p* < 0.001), suggesting a link between hepatic lipid/bile acid metabolism and pulmonary hemodynamics [54].

Emerging evidence suggests that targeting bile acid signaling may offer therapeutic potential in PAH. For example, FXR agonists have been shown to reduce hepatic inflammation and fibrosis in preclinical models, while TGR5 activation improves endothelial function and reduces vascular remodeling. Although direct studies in PAH are limited, the observed cholestatic profile in PAH patients supports the involvement of bile acid pathways in disease progression [55]. Future research should explore whether modulation of FXR/TGR5 signaling can ameliorate pulmonary vascular dysfunction in PAH, particularly in patients with concurrent liver disease or cholestasis.

### 3.3. Skeletal Muscle: Energy Crisis and Wasting

#### 3.3.1. Muscle Atrophy and Release of Metabolic Signals

Skeletal muscle wasting in pulmonary arterial hypertension (PAH) is not merely a loss of muscle mass, but an active metabolic event characterized by enhanced catabolism and the systemic release of signaling molecules. This atrophy, affecting up to 60% of patients and linked to increased mortality, is driven by molecular pathways that shift the balance from protein synthesis to degradation [17,18]. A central mediator is growth differentiation factor 11 (GDF11), whose elevated serum levels in PAH patients activate signal transducer and activator of transcription 3 (STAT3) phosphorylation [56]. In experimental models, this STAT3 activation promotes proteolytic pathways, including the induction of key muscle-specific E3 ubiquitin ligases atrogin-1 and muscle RING-finger protein-1 (MuRF1), which tag proteins for destruction via the ubiquitin proteasome system [17,56]. Furthermore, pharmacological inhibition of STAT3 in animal models partially reverses muscle wasting, highlighting its pathogenic role and potential as a therapeutic target [56].

The atrophy process releases a cascade of metabolites and signaling molecules with systemic effects. The breakdown of muscle proteins, particularly myofibrillar components, releases free amino acids. Among these, branched-chain amino acids (BCAAs), such as leucine, isoleucine, and valine, serve not only as substrates but also as potent metabolic signals. They can modulate Mechanistic Target of Rapamycin (mTORC1) activity, a master regulator of protein synthesis, although in the PAH catabolic state, this anabolic signaling is often blunted [17]. Concurrently, muscle-derived inflammatory cytokines, such as tumor necrosis factor-α (TNF-α) and interleukin-6 (IL-6), are elevated and correlate with the expression of atrophy-inducing factors like myostatin [57]. Myostatin itself, a negative regulator of muscle growth, is significantly increased in PAH serum and further propagates the atrophic signal locally and possibly systemically [57,58]. This creates a vicious cycle: systemic inflammation and metabolic stress (e.g., from hypoxemia) drive muscle catabolism, which in turn releases factors that may exacerbate pulmonary vascular remodeling and systemic metabolic dysregulation, thereby forming a muscle–pulmonary axis of disease progression [56,59].

#### 3.3.2. The Metabolic Roots of Exercise Intolerance

Exercise intolerance in PAH is fundamentally rooted in a profound energy crisis within skeletal muscle, arising from multi-level dysfunction in energy production, substrate utilization, and demand–supply matching, creating a vicious cycle with whole-body energy metabolism.

- Mitochondrial Dysfunction and Oxidative Capacity Impairment:

The primary defect lies in impaired ATP regeneration via oxidative phosphorylation (OXPHOS). In vivo studies using ^31^P-magnetic resonance spectroscopy (^31^P-MRS) reveal delayed post-exercise phosphocreatine (PCr) recovery and abnormal pH regulation in PAH muscle, indicating compromised oxidative metabolism and bioenergetic inefficiency [60]. While intrinsic mitochondrial complex function may be preserved, the assembly of mitochondrial supercomplexes—essential for efficient electron transfer and respiratory control—is disrupted, particularly in oxidative type I fibers [61]. This structural disorganization is compounded by altered mitochondrial dynamics, specifically reduced expression of Mitofusin 2 (Mfn2), a protein critical for mitochondrial fusion and network integrity. Impaired fusion leads to fragmented, less efficient mitochondria, further reducing the muscle’s oxidative capacity [17].

- Metabolic Reprogramming and Glycolytic Shift:

In response to chronic energy stress and possibly hypoxia, PAH skeletal muscle undergoes a metabolic shift from oxidative towards glycolytic metabolism. This is evidenced by an increased ratio of glycolytic (phosphofructokinase, PFK) to oxidative (3-hydroxyacyl-CoA dehydrogenase, 3-HAD) enzyme activity [62]. The activity of citrate synthase, a key enzyme in the tricarboxylic acid (TCA) cycle, is also reduced and correlates with a lower anaerobic threshold during exercise [62]. Hypoxia-inducible factors stabilize under reduced oxygen tension and upregulate pyruvate dehydrogenase kinase (PDK), which inhibits the entry of pyruvate into the TCA cycle, effectively shunting glucose-derived carbons away from efficient ATP production and towards lactate generation [63]. This metabolic reprogramming results in early reliance on inefficient glycolysis during exercise, leading to premature fatigue and lactate accumulation.

- Fiber Type Transition and Perfusion Deficit:

The metabolic crisis is exacerbated by a shift in muscle fiber composition from slow-twitch, oxidative, fatigue-resistant type I fibers to fast-twitch, glycolytic, fatigable type II fibers [17,62]. This shift, driven in part by the transcription factor FoxO1, reduces the muscle’s inherent oxidative capacity [64]. Furthermore, impaired muscle perfusion due to reduced capillarity critically limits oxygen and nutrient delivery. This microvascular rarefaction is linked to the downregulation of the pro-angiogenic microRNA miR-126, which disinhibits its target SPRED-1 and suppresses VEGF signaling, thereby impairing angiogenesis [65]. The reduced capillary density strongly correlates with peak oxygen uptake (VO_2max_), underscoring its role in limiting oxidative metabolism [65].

**Figure 2:**
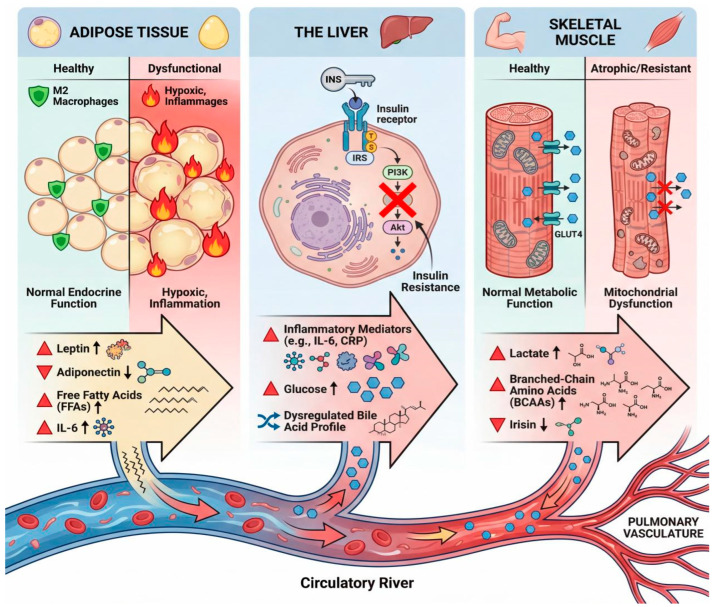
Metabolic dysfunction of peripheral organs contributes a pathogenic “secretome” in PAH. (**Left**) Adipose Tissue Dysfunction: Inflamed, hypoxic adipose tissue shifts from an anti-inflammatory endocrine organ to a pro-inflammatory factory. It secretes increased levels of leptin and pro-inflammatory cytokines (e.g., IL-6) and free fatty acids (FFAs), while protective adiponectin production decreases. (**Middle**) Hepatic Insulin Resistance: The insulin-resistant liver exhibits blunted insulin signaling (via IRS/PI3K/Akt) and becomes a source of inflammatory mediators (e.g., IL-6, CRP), excess glucose, and an altered bile acid profile, all contributing to systemic metabolic inflammation. (**Right**) Skeletal Muscle Wasting and Dysmetabolism: Muscle atrophy and mitochondrial dysfunction are hallmarks of PAH. This leads to increased lactate production, release of branched-chain amino acids (BCAAs), and potentially decreased myokine (e.g., irisin) secretion, reflecting and exacerbating systemic catabolism and energy crisis. Collectively, these organ-specific alterations flood the circulation with factors that target the pulmonary vasculature.

This constellation of defects—impaired mitochondrial efficiency, a glycolytic metabolic shift, fiber type transition, and microvascular insufficiency—creates a self-perpetuating cycle. The muscle’s inability to produce energy efficiently leads to early fatigue and exercise limitation (reduced “demand”). This physical inactivity, in turn, promotes further deconditioning, aggravating muscle atrophy and metabolic dysfunction. Simultaneously, signals from the wasting muscle (e.g., metabolites, myokines) and the compromised cardiac output in PAH contribute to systemic metabolic disturbances, such as insulin resistance and increased systemic inflammation, which further feed back to worsen skeletal muscle health [66,67]. Thus, the “energy crisis” in PAH skeletal muscle is both a cause and a consequence of a broader, systemic metabolic disorder, forming the core of exercise intolerance (Figure 2).

## 4. Immunometabolic Reprogramming: The Fuel and Engine of Inflammation

### 4.1. Macrophages: Metabolic Polarization in PAH

In PAH, macrophages undergo profound metabolic reprogramming that dictates their functional polarization and contributes decisively to vascular inflammation and remodeling. This metabolic shift is not merely an epiphenomenon but a central mechanism driving disease progression, making macrophage metabolism a critical focus for understanding PAH pathophysiology and developing targeted therapies.

#### 4.1.1. Glycolytic Dependency in Pro-Inflammatory (M1) Polarization

In PAH, macrophages frequently polarize towards a pro-inflammatory M1 phenotype, a process fueled by a metabolic switch to aerobic glycolysis, akin to the Warburg effect observed in cancer cells. This glycolytic dependency supports the bioenergetic and biosynthetic demands of intense inflammatory activity. M1 macrophages exhibit a truncated tricarboxylic acid (TCA) cycle and dampened mitochondrial oxidative phosphorylation (OXPHOS), leading to accumulation of metabolic intermediates that stabilize hypoxia-inducible factor-1α (HIF-1α). HIF-1α, in turn, transcriptionally upregulates glycolytic enzymes and promotes a sustained glycolytic flux [68]. In vitro studies using lipopolysaccharide (LPS)-stimulated macrophages—a model of acute inflammation—have demonstrated a substantial metabolic shift, including a 2.3-fold increase in lactate production and a reduction in oxidative phosphorylation (OXPHOS) compared to resting cells [69]. While these quantitative data are derived from non-PAH models, they illustrate the profound metabolic reprogramming that underpins pro-inflammatory macrophage activation. In the context of PAH, macrophages isolated from hypoxic rodent models show a 3.2-fold increase in inducible nitric oxide synthase (iNOS) activity [70], and clinical studies have reported that the strength of this glycolytic signature correlates with disease severity, as patients with high glycolytic macrophage activity exhibit a 2.1-fold higher risk of right ventricular failure [69].

This metabolic configuration is intrinsically linked to inflammatory output. This glycolytic dependency supports rapid ATP production and provides biosynthetic precursors for cytokine synthesis. Importantly, disrupted mitochondrial metabolism in M1 macrophages leads to electron leakage and ROS generation, which activate the NLRP3 inflammasome and promote secretion of IL-1β and TNF-α [71,72]. Moreover, accumulated succinate from a truncated TCA cycle stabilizes HIF-1α and directly enhances IL-1β transcription, establishing a positive feedback loop between metabolism and inflammation [73]. In the context of PAH, this M1–glycolysis axis is pathological. Macrophages isolated from hypoxic PAH models show a 3.2-fold increase in inducible nitric oxide synthase (iNOS) activity, an enzyme linked to glycolysis and nitric oxide production, which further contributes to vascular injury and smooth muscle cell proliferation [70]. The strength of this glycolytic signature correlates with disease severity, as patients with high glycolytic macrophage activity exhibit a 2.1-fold higher risk of right ventricular failure [74]. Furthermore, initial hypoxic stimulation may induce “trained immunity” in macrophages via epigenetic modifications, leading to persistent alterations in their metabolic and inflammatory response programs. This implies that macrophages may maintain a highly active pro-inflammatory state even after the stimulus is removed, providing a novel theoretical framework for explaining the chronic and refractory inflammation in PAH [75].

#### 4.1.2. Altered Substrate Utilization: Iron and Fatty Acid Metabolism

Beyond glucose, the polarization of macrophages in PAH involves significant reprogramming of other substrate utilization pathways, notably iron and fatty acid metabolism, which further fine-tune their inflammatory and fibrotic functions. These alterations not only meet the energy and biosynthetic demands of the cells but, more critically, alter the composition of the intracellular metabolite pool. These metabolites themselves can act as signaling molecules regulating polarization status, inflammatory signaling, and the epigenetic landscape.

Iron metabolism is a key modulator of macrophage polarization. Iron-laden macrophages tend to adopt an M1-like pro-inflammatory state. Intracellular iron overload promotes ROS generation via the Fenton reaction, which stabilizes HIF-1α and perpetuates glycolysis, creating a feed-forward loop for M1 activation. In vitro studies have demonstrated that iron loading of macrophages enhances pro-inflammatory cytokine production, including a 2.1-fold increase in IL-6 release and a 1.8-fold upregulation of iNOS [76]. Although direct evidence in PAH patients is limited, these findings suggest that dysregulated iron metabolism within perivascular macrophages could contribute to oxidative stress and endothelial dysfunction, potentially fueling vascular remodeling in the human disease.

Fatty acid metabolism delineates the functional dichotomy between M1 and M2 macrophages. While M1 macrophages downregulate fatty acid oxidation (FAO), anti-inflammatory and pro-fibrotic M2 macrophages rely heavily on OXPHOS fueled by FAO. This metabolic profile supports their long-term tissue residency and functions in repair and fibrosis. Studies of macrophage polarization have demonstrated that IL-4-induced M2 differentiation is associated with metabolic remodeling, including upregulation of FAO-related genes such as Cpt1a (by approximately 1.8-fold) and a increase in mitochondrial mass [77]. These findings provide a framework for understanding how metabolic reprogramming may sustain M2-like phenotypes in the PAH vasculature. The nuclear receptor PPARγ is a master regulator of this program, promoting FAO and M2 differentiation [78]. In PAH, the balance between these states is crucial. While early M2 activity might be reparative, sustained or excessive M2 polarization, particularly in conditions like connective tissue disease-associated PAH (CTD-PAH), is associated with pathological fibrosis. CTD-PAH patients exhibit a 2.3-fold increase in M2 macrophages (CD206+) in lung lesions, correlating with worse outcomes [79]. Furthermore, disruption of metabolites like α-ketoglutarate (α-KG), which is essential for M2 polarization, can skew macrophages toward a pro-inflammatory phenotype. Infection with Porphyromonas gingivalis reduces the α-KG/succinate ratio by 60% and suppresses M2 marker expression by 45% [80].

#### 4.1.3. Therapeutic Potential of Targeting Macrophage Metabolism

The centrality of metabolic reprogramming in macrophage dysfunction presents a compelling therapeutic avenue for PAH. The goal is to reverse the pathological polarization, either by inhibiting the fuel of inflammation (e.g., glycolysis in M1) or by promoting a beneficial metabolic state (e.g., FAO/OXPHOS in M2). Future strategies are evolving toward more precise and mechanism-based approaches, fully leveraging the dual role of metabolites—as both targets and therapeutic agents.

- Precision Targeting of Glycolysis:

Beyond broad-spectrum glycolytic inhibitors like 2-deoxyglucose (2-DG) and 3-bromopyruvate (3-BrPA), emerging research is focusing on more specific nodes of glycolytic regulation. For example, targeting the key regulatory enzyme PFKFB3 (6-phosphofructo-2-kinase/fructose-2,6-bisphosphatase 3), which controls the “throttle” of glycolytic flux [81,82]. In preclinical models of inflammatory disease (e.g., LPS-induced lung injury and cancer models), specific PFKFB3 inhibitors (e.g., PFK158) have demonstrated the ability to selectively suppress glycolysis in M1 macrophages while potentially reducing off-target effects on normal cells [83,84,85]. These findings provide a rationale for investigating PFKFB3 inhibition in experimental PAH models and, if successful, ultimately in patients.

- Modulating Metabolite Signaling:

Therapeutic strategies are not limited to pathway inhibition but also include harnessing or mimicking metabolites with anti-inflammatory properties. For instance, M1 macrophages synthesize large amounts of itaconate during inflammation, a product catalyzed by the enzyme encoded by the ACOD1 (IRG1) gene [86]. Itaconate and its derivatives can activate the antioxidant transcription factor NRF2 by alkylating the KEAP1 protein and inhibit the production of pro-inflammatory cytokines, acting as a negative feedback regulator [87,88]. Therefore, enhancing this endogenous anti-inflammatory braking system through drug delivery of itaconate derivatives or small-molecule activators provides a novel “metabolo-immunomodulatory” approach for PAH treatment [89].

- Addressing Mitochondrial Dysfunction:

Impaired mitochondrial function is a feature of PAH macrophages. Therapies aimed at restoring mitochondrial health, such as cannabidiol (CBD), can rebalance metabolism. CBD treatment restores OXPHOS capacity by 30% and reduces ROS production by 40% in PAH macrophages, leading to improved vascular pathology [90,91]. This approach highlights that therapeutic success may not only involve inhibiting a pathogenic pathway but also restoring a healthy metabolic phenotype.

- Advanced Monitoring with Metabolic Imaging:

Evaluating the efficacy of such targeted metabolic therapies requires corresponding tools [92,93]. Beyond traditional FDG-PET (reflecting glucose uptake), cutting-edge technologies like Hyperpolarized 13C-Pyruvate Magnetic Resonance Spectroscopy (13C-MRS) enable non-invasive, real-time monitoring of flux through specific metabolic pathways in vivo (e.g., the conversion rate of pyruvate to lactate) [94,95,96], thereby directly quantifying the pharmacodynamic effects of glycolytic inhibitors. This will provide crucial closed-loop feedback for realizing personalized metabolic therapy in PAH [96].

In conclusion, macrophage metabolic polarization is a dynamic and druggable axis in PAH. The understanding of metabolic reprogramming has deepened from a simple “energy switch” to a “signaling hub,” offering more diverse targets for intervention. Future therapeutic strategies will likely combine traditional vasodilators with precise metabolo-immunomodulators, personalized based on patient-specific macrophage metabolic profiles, while utilizing advanced metabolic imaging for real-time efficacy monitoring, to more effectively halt or reverse the disease process.

### 4.2. Lymphocytes: Metabolic Basis of Subset Imbalance

Lymphocyte metabolism plays a pivotal role in determining subset differentiation and function in pulmonary arterial hypertension (PAH). Metabolic reprogramming within T cells drives the imbalance between pro-inflammatory and regulatory subsets, contributing directly to vascular inflammation and remodeling.

#### 4.2.1. Metabolism-Driven Th17/Treg Dysregulation

The balance between T helper 17 (Th17) cells and regulatory T cells (Tregs) is critically regulated by their distinct metabolic programs. In PAH, a shift toward glycolytic metabolism promotes the differentiation and expansion of pro-inflammatory Th17 cells, while impairing the suppressive function of Tregs that rely more on oxidative phosphorylation.

Th17 differentiation is fueled by increased glycolysis. In idiopathic PAH (iPAH), CD4+ T cells exhibit a 2-fold higher basal glycolysis rate compared to controls (*p* < 0.05) [97]. This glycolytic shift is driven, in part, by the overexpression of aldehyde dehydrogenase 1A3 (ALDH1A3), which increases glycolytic flux by 30% through acetyl-CoA production, linking metabolic reprogramming to epigenetic regulation that sustains the Th17 phenotype [98]. Additionally, the kynurenine pathway—elevated in PAH patients with plasma levels of 2.8 μmol/L vs. 1.9 μmol/L in controls (*p* < 0.001)—modulates this balance. Kynurenine, produced via indoleamine 2,3-dioxygenase (IDO) upregulated by IL-6, reduces Th17 differentiation by 40% in vitro while enhancing Treg suppressive capacity by 50% (*p* < 0.05) [99]. However, in PAH, despite elevated kynurenine, Treg function is compromised due to concurrent mitochondrial dysfunction.

Treg function and stability are highly dependent on mitochondrial metabolism. iPAH patients show a 40% reduction in mitochondrial transmembrane potential in CD8+ T cells (*p* < 0.05), a defect that extends to Tregs, impairing their oxidative phosphorylation [100]. This mitochondrial dysfunction is exacerbated by metabolic signals such as leptin, which is 2.5-fold higher in iPAH patients. In vitro studies have demonstrated that leptin, which is elevated 2.5-fold in iPAH patients, can directly inhibit mitochondrial complex I in Tregs, reducing their suppressive capacity by approximately 40% [101]. Converging with this mechanistic evidence, cross-sectional analyses of iPAH patients have revealed an expansion of non-suppressive CD4+CD45RA-FoxP3low Tregs compared to healthy controls (4.06% vs. 2.79%, *p* < 0.01) [102]. These complementary lines of evidence—from both experimental models and human samples—suggest that both circulating factors and intrinsic Treg abnormalities contribute to immune dysregulation in PAH. The resultant Th17/Treg imbalance, quantified by a reduced ratio in certain subtypes (e.g., 1.2 in CHD-PAH vs. 2.8 in iPAH, *p* < 0.05), drives vascular pathology through IL-17-mediated PASMC proliferation and impaired immunoregulation [103]. Collectively, these findings, derived from cross-sectional and case–control studies of varying sample sizes, support a central role for metabolic dysregulation in T-cell imbalance in PAH. However, the quantitative relationships (e.g., fold-changes) should be interpreted in the context of each study’s design and population [97,99,102].

#### 4.2.2. Brief Notes on Other Immune Cells (Neutrophils, B Cells)

While T lymphocytes are central, other immune cells contribute to PAH pathophysiology through distinct metabolic adaptations. Neutrophils in PAH exhibit a hyperinflammatory phenotype supported by glycolytic metabolism. An elevated neutrophil-to-lymphocyte ratio (NLR) > 2.54 is associated with a 21% in-hospital mortality in chronic thromboembolic PH (CTEPH) patients (*p* < 0.05) [104]. In PAH, the highest NLR tertile correlates with a 3-fold increase in mortality (*p* < 0.05) and associates with disease severity (NYHA class, r = 0.5; BNP, r = 0.4; *p* < 0.001) [105]. Metabolic studies suggest that neutrophil glycolytic activity fuels oxidative burst and neutrophil extracellular trap (NET) formation, perpetuating vascular injury and inflammation, though detailed metabolic profiling in PAH remains an area for further study [106,107].

B cells contribute to PAH through antibody production and cytokine secretion, processes that are metabolically demanding. B cell activation and differentiation into antibody-secreting plasmablasts require increased glycolysis and glutaminolysis [108,109]. In PAH, perivascular B cell infiltration is observed in models of C-C Chemokine Receptor Type 7 (CCR7) deficiency, associated with a 35% increase in pulmonary vascular muscularization [110,111]. Furthermore, autoantibodies against endothelial cells are detected in a subset of iPAH patients, which may enhance T cell activation by 30% (*p* < 0.05) [112,113]. B cell metabolism in PAH, particularly its interplay with T follicular helper (Tfh) cells and the impact of metabolic inhibitors (e.g., JAK inhibitors like ruxolitinib, which reduce plasmablast differentiation by 40% [114]), represents an emerging frontier in understanding immune-metabolic dysregulation.

**Figure 3:**
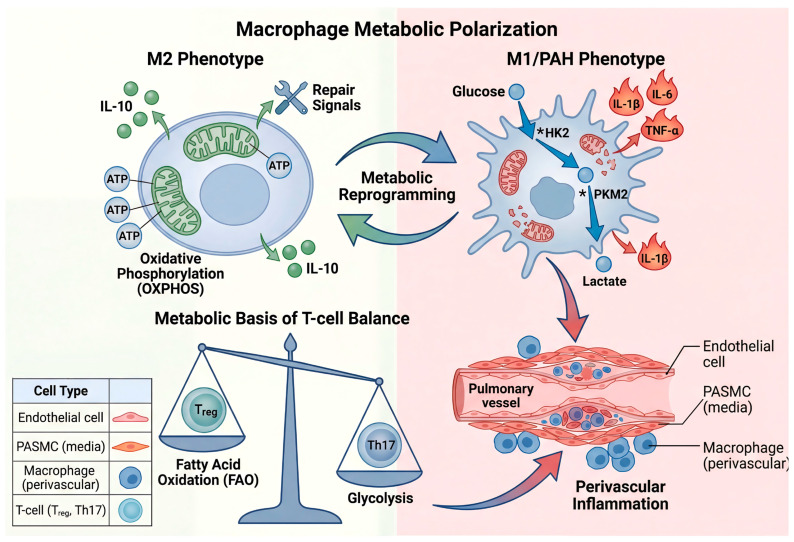
Immunometabolic reprogramming fuels inflammation in PAH. (**Left**) Macrophage Polarization is Driven by Metabolic Shifts: In PAH, macrophages undergo a metabolic switch from oxidative phosphorylation (OXPHOS) to aerobic glycolysis (the “Warburg effect”), a process known as immunometabolic reprogramming. This fuels a pro-inflammatory (M1-like) phenotype, characterized by increased expression of glycolytic enzymes (HK2, PKM2) and the release of potent cytokines (IL-1β, IL-6, TNF-α). In contrast, anti-inflammatory (M2-like) macrophages rely on OXPHOS and produce resolving factors like IL-10. (**Right**) T Cell Fate is Linked to Metabolic Substrate Utilization: The balance between pro-inflammatory T helper 17 (Th17) cells and anti-inflammatory regulatory T (Treg) cells is metabolically governed. Th17 differentiation is supported by glycolysis, whereas Treg function depends on fatty acid oxidation (FAO). In PAH, this balance is skewed towards the pro-inflammatory Th17 state. These metabolically activated immune cells infiltrate the perivascular space, creating a chronic inflammatory milieu that directly promotes vascular remodeling.

In summary, lymphocyte subset imbalance in PAH is deeply rooted in cellular metabolism, with Th17/Treg dysregulation driven by glycolytic expansion and mitochondrial compromise. Neutrophils and B cells further contribute through their own metabolic programs, highlighting a broad immuno-metabolic network that sustains pulmonary vascular disease. Targeting these metabolic pathways offers a promising strategy for restoring immune homeostasis in PAH (Figure 3).

## 5. The Convergence: Synergy Between Systemic Metabolism and Immunometabolism

### 5.1. Insulin Resistance/Hyperglycemia as a Central Hub

Insulin resistance and hyperglycemia constitute a central pathogenic hub in PAH, creating a critical interface between systemic metabolic dysfunction and sustained immune activation [12,13,115]. The hyperglycemic milieu not only promotes direct vascular injury via advanced glycation end products (AGEs) but also establishes a pro-inflammatory state by priming both innate and adaptive immune cells [116]. Epidemiological and clinical studies indicate a high prevalence of insulin resistance in PAH, with reports suggesting it affects approximately 51.4% of patients, although its direct correlation with disease severity remains a subject of debate [9,10]. At a mechanistic level, hyperglycemia has been shown to activate inflammatory transcription factors such as NF-κB in endothelial cells and monocytes, thereby promoting vascular inflammation [116]. In the context of PAH, CD4^+^ T cells from patients exhibit a shift toward glycolysis, and pathway analysis suggests that this metabolic reprogramming may be modulated by the mTOR signaling axis, which links nutrient sensing to inflammatory cytokine production [117]. However, direct causal evidence for mTOR-driven T-cell dysfunction in PAH remains to be established. Thus, insulin resistance acts as both a consequence and a driver of the immunometabolic dysregulation that fuels PAH progression, highlighting it as a potential target for dual-pathway intervention.

### 5.2. Circulating Metabolites as Signaling Messengers

Beyond their roles as energy substrates or waste products, specific circulating metabolites function as potent signaling molecules that directly connect systemic metabolism to pulmonary vascular and immune cell biology in PAH. Mechanistically, in vitro studies have demonstrated that lactate can promote M2-like macrophage polarization via monocarboxylate transporter 1 (MCT1), which in turn enhances pulmonary artery smooth muscle cell (PASMC) proliferation [118]. Clinically, elevated plasma lactate levels (2.7 ± 0.5 mmol/L in PAH vs. 1.2 ± 0.3 mmol/L in controls) are associated with exercise intolerance and may serve as a marker of disease severity [118]. Furthermore, disruptions in TCA cycle intermediates, such as reduced oxaloacetate levels, have been associated with enhanced pro-inflammatory cytokine production by peripheral blood mononuclear cells (PBMCs) in studies of systemic inflammation [119,120,121]. This suggests that similar metabolic disturbances could contribute to immune dysregulation in PAH, although direct evidence in PAH patients is still needed. Specific long-chain fatty acid metabolites (e.g., acylcholines) serve as early biomarkers in SSc-PAH and act as ligands for G protein-coupled receptor 40/Free Fatty Acid Receptor 1 (GPR40/FFAR1) on PASMCs, driving proliferative and oxidative stress responses [122,123]. These findings underscore the paradigm where metabolites serve as essential communicators, translating systemic metabolic imbalances into local pathogenic signals within the pulmonary vasculature.

### 5.3. The Organ Crosstalk Network: The Adipose–Liver–Immune–Lung Axis

**Figure 4:**
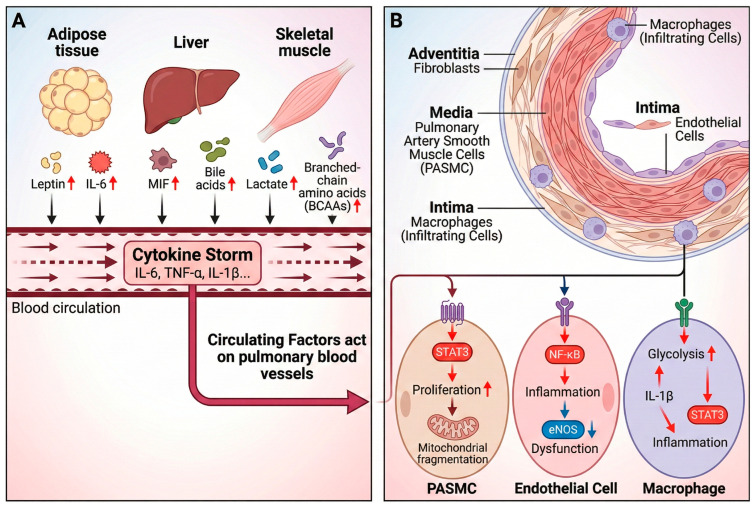
Converging systemic signals drive pulmonary vascular remodeling. (**A**) Systemic compartment. Dysfunctional peripheral organs (adipose tissue, liver, skeletal muscle) and activated immune cells release a barrage of circulating mediators, including adipokines (e.g., leptin), hepatokines (e.g., MIF), and pro-inflammatory cytokines (e.g., IL-6, TNF-α). This systemic overflow contributes to a “Cytokine Storm” that bathes the pulmonary vasculature. (**B**) Cell-intrinsic responses in the pulmonary vasculature. Circulating factors bind to receptors on pulmonary artery smooth muscle cells (PASMCs), endothelial cells, and infiltrating macrophages, triggering intracellular signaling cascades (e.g., STAT3, NF-κB). This leads to metabolic reprogramming (glycolytic shift, mitochondrial fragmentation) and a pathological phenotype of proliferation, apoptosis resistance, and sustained inflammation. Abbreviations: IL-6, interleukin-6; TNF-α, tumor necrosis factor-alpha; STAT3, signal transducer and activator of transcription 3; NF-κB, nuclear factor kappa-light-chain-enhancer of activated B cells. The red upward arrow indicates up-regulation.

The pathophysiology of PAH is increasingly recognized as a disorder of inter-organ communication, with the “adipose–liver–immune–lung axis” representing a dynamic, self-amplifying network. Dysfunctional adipose tissue in PAH contributes to systemic lipotoxicity and inflammation by releasing excess free fatty acids (FFAs) and adipokines. These signals impair hepatic metabolism, leading to dyslipidemia characterized by low HDL and high triglyceride levels, which are associated with worse right ventricular (RV) outcomes [20]. The inflamed liver, in turn, contributes to the systemic inflammatory milieu by producing acute-phase proteins and complement components. Notably, complement C3a is elevated 2.8-fold in PAH plasma and is associated with disease progression, demonstrating how hepatic-derived immune activators can remotely fuel pulmonary vascular inflammation [124]. This axis is perpetuated by immune cells, which are metabolically reprogrammed by these systemic cues. For example, M2 macrophages, whose prevalence is linked to pulmonary vascular resistance, may be sustained by both adipose-derived signals and liver-produced factors [125,126]. Furthermore, metabolic disturbances in one organ can directly impact another, as seen in the correlation between low plasma oxaloacetate levels and an increased risk of RV failure [121]. This intricate crosstalk validates PAH as a systemic disease and argues for therapeutic strategies that target the interconnected metabolic and immune dialogues across multiple organs (Figure 4).

## 6. Clinical Translation and Therapeutic Perspectives

### 6.1. Biomarkers for Diagnosis and Phenotyping: Integrating Systemic Metabolic and Immunometabolic Profiles

Advancing the diagnostic and prognostic toolkit for PAH necessitates the development of integrated biomarker panels that capture both systemic metabolic derangements and immune dysregulation. Beyond conventional hemodynamic and functional assessments, the incorporation of specific metabolic ratios—such as the adiponectin/leptin ratio (reflecting adipose tissue health and systemic inflammation)—and patterns of circulating metabolites like branched-chain amino acids (indicative of muscle catabolism) could significantly refine patient stratification [9,10]. These systemic markers should be coupled with immunometabolic biomarkers that directly reflect pathogenic activity. For instance, cross-sectional and cohort studies have reported that elevated plasma levels of complement C3a (2.8-fold higher than controls) are associated with clinical worsening in PAH, while increased IL-32 levels correlate with higher mean pulmonary arterial pressure in systemic sclerosis-associated PAH (SSc-PAH) [124,125]. These findings highlight their potential utility as prognostic biomarkers, although their role as causal drivers of disease progression requires further investigation. Furthermore, metabolomic signatures, including elevated kynurenine (a 2.7-fold increase linked to T cell activity) and specific long-chain fatty acid acylcholines (which can predict PAH onset up to two years prior to diagnosis with an AUC of 0.86), offer dynamic, mechanism-based insights into disease progression [25,122,127]. The integration of these multi-omic profiles via bioinformatics and machine learning holds promise for defining distinct metabolic endophenotypes, moving PAH classification towards a biology-driven framework that can guide personalized therapeutic interventions [25,128] (Table 2).

### 6.2. Repurposing and Novel Therapeutic Strategies

#### 6.2.1. Antidiabetic Agents in PAH (SGLT2i, GLP-1 RAs, Metformin)

The repurposing of antidiabetic drugs presents a rational strategy for PAH treatment, given the disease’s underpinnings in insulin resistance and inflammatory metabolism. Given the central role of insulin resistance and inflammatory metabolism in PAH, the repurposing of antidiabetic agents represents a rational therapeutic strategy [129,130]. Although direct clinical trial evidence in PAH populations is still forthcoming, the well-established cardiorenal protective mechanisms of sodium-glucose cotransporter-2 inhibitors (SGLT2i) and glucagon-like peptide-1 receptor agonists (GLP-1 RAs) provide a strong rationale for their investigation in alleviating right ventricular (RV) afterload and dysfunction [131,132]. Targeted clinical trials are warranted to determine whether these agents can improve functional capacity or hemodynamics in PAH patients, particularly those with overt metabolic syndrome [9,10]. Metformin, an activator of AMP-activated protein kinase (AMPK), could directly counteract the pathological glycolytic shift (“Warburg effect”) observed in PAH vascular cells and cardiomyocytes by promoting glucose oxidation and improving insulin sensitivity [9,21,46]. Its potential to modulate immune cell metabolism and inhibit PASMC proliferation aligns with the need to disrupt the metabolic–inflammatory axis [42,117]. Targeted clinical trials are warranted to evaluate whether these agents can improve functional capacity (e.g., 6 min walk distance), hemodynamics, or RV function in PAH patients, particularly in subgroups with overt metabolic syndrome [9,10].

#### 6.2.2. Anti-Inflammatory and Direct Immunometabolic Modulators

Moving beyond broad immunosuppression, direct pharmacological targeting of immunometabolic nodes offers a precision approach to disrupt the PAH vicious cycle. In addition to biologics against specific cytokines like IL-6 or IL-1β, small molecules that modulate the metabolic machinery of immune cells are of great interest. Preclinical evidence suggests that inhibitors of pyruvate kinase M2 (PKM2), a key glycolytic enzyme and transcriptional coactivator for HIF-1α, may simultaneously dampen excessive glycolysis in vascular cells and impair pro-inflammatory M1 macrophage polarization [22,46]. Similarly, dihydroorotate dehydrogenase (DHODH) inhibitors, which block de novo pyrimidine synthesis, have shown potential in targeting the hyper-proliferative state of lymphocytes implicated in PAH immunopathology in early experimental models [7,117]. These concepts, while promising, require rigorous validation in vivo and in clinical settings. These approaches are complemented by agents targeting specific crosstalk mechanisms, such as inhibitors of monocarboxylate transporters (MCTs) to block lactate-facilitated macrophage M2 polarization, or mTOR inhibitors like rapamycin to reduce pathogenic T cell glycolysis and vascular remodeling [22,117,118]. The therapeutic goal is to break the self-amplifying loop wherein dysregulated metabolism fuels inflammation and vice versa.

#### 6.2.3. Lifestyle Interventions: Nutrition, Diet, and Exercise Revisited

A reconceptualization of PAH as a systemic metabolic–immune disorder elevates the importance of foundational lifestyle interventions. Nutritional strategies must address the common catabolic state and specific deficiencies, such as iron deficiency (present in ~40% of patients), as correction with iron supplementation has been shown to improve exercise capacity [24]. Investigation into optimal dietary patterns is needed: While a ketogenic diet’s provision of ketones as an alternative fuel is theoretically appealing for the energy-starved RV [133,134], its systemic and pulmonary vascular effects in PAH remain entirely unexplored. This hypothesis warrants careful investigation before any clinical recommendations can be made. In contrast, diets with established benefits for insulin sensitivity and inflammation, such as the Mediterranean diet, hold more immediate and evidence-based promise. Diets that improve insulin sensitivity and reduce inflammation (e.g., Mediterranean diet) hold more immediate promise. Supervised, personalized exercise rehabilitation must be valued not only for improving cardiopulmonary fitness but for its profound capacity to remodel systemic metabolism—enhancing skeletal muscle mitochondrial function, insulin sensitivity, and the release of anti-inflammatory myokines [9,135]. These non-pharmacological interventions, grounded in a deep metabolic rationale, should be integrated as essential components of a comprehensive PAH management plan (Table 3).

### 6.3. Future Directions: Targeted Delivery, Patient Stratification, and the Microbiome–Metabolism Axis

**Figure 5:**
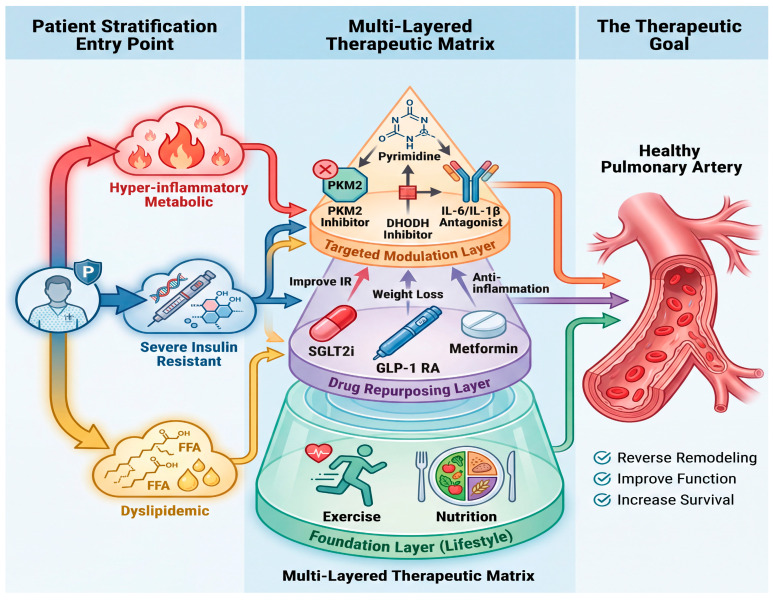
Therapeutic implications: a multi-pronged strategy targeting the systemic metabolic–immune axis. Future management of PAH may evolve from solely targeting the pulmonary vasculature to a precision medicine approach that also addresses upstream systemic drivers. (**Left**) Patient Stratification: Identification of dominant pathobiological subphenotypes (e.g., “hyper-inflammatory metabolic,” “severe insulin resistant”) could guide therapy selection. (**Middle**) Multi-Layer Interventions: Therapeutic strategies can be envisioned across several levels: Lifestyle Foundation: Structured exercise rehabilitation and tailored nutritional support to improve metabolic health. Drug Repurposing: Utilizing agents like SGLT2 inhibitors, GLP-1 receptor agonists, and metformin to improve insulin sensitivity, reduce weight, and exert pleiotropic anti-inflammatory effects. Targeted Immunometabolic Modulators: Novel agents directly targeting key nodes in immunometabolism (e.g., PKM2, DHODH) or specific cytokines (e.g., IL-6, IL-1β) to reprogram immune cell function. (**Right**) Therapeutic Goal: The aim of this integrated strategy is to disrupt the systemic drivers of disease, thereby promoting reverse vascular remodeling, improving cardiopulmonary function, and ultimately enhancing patient survival.

The trajectory of PAH therapy points toward greater personalization and systemic integration. First, advancing drug delivery through organ- or cell-specific targeting (e.g., ligand-conjugated nanoparticles) could maximize the efficacy and safety of potent metabolic or immunomodulatory agents, particularly for delivery to the pulmonary vasculature and RV [46,136]. Second, patient stratification will evolve from clinical parameters to deep, multi-omic phenotyping. Integrating data from single-cell analyses, plasma metabolomics (e.g., as in [127]), and proteomics can identify dominant driver pathways, classifying patients into subgroups such as “glycolytic-dominant,” “lipotoxic,” or “hyper-inflammatory” phenotypes for tailored therapy [128,137]. Third, modulation of the gut microbiome–metabolism axis emerges as a novel therapeutic frontier. Third, modulation of the gut microbiome–metabolism axis emerges as a novel therapeutic frontier. Emerging preclinical evidence indicates that altering gut microbiota can attenuate experimental PH, likely mediated by microbial metabolites like short-chain fatty acids which influence host immune and metabolic homeostasis [138]. However, clinical translation of these findings remains in its infancy. Strategies such as targeted probiotics, prebiotics, or fecal microbiota transplantation represent intriguing but speculative avenues that warrant further exploration as adjunctive therapies. These forward-looking approaches, combining precision diagnostics, repurposed and novel therapeutics, and holistic lifestyle modulation, chart a course toward truly personalized and effective management of PAH (Figure 5).

## 7. Conclusions

### 7.1. Recapitulation: PAH as a Systemic Network Disorder

The evidence synthesized in this review establishes that pulmonary arterial hypertension (PAH) is far from a disease confined to the pulmonary vasculature. Rather, it is fundamentally a systemic metabolic–immunological network disorder whose most visible endpoint is pathological pulmonary vascular remodeling. The evolution of historical perspectives has revealed a widespread “Warburg effect”-like metabolic reprogramming—characterized by enhanced glycolysis and suppressed mitochondrial oxidative phosphorylation—that is present not only in pulmonary vascular cells but also in right ventricular cardiomyocytes and skeletal muscle, indicating a whole-body metabolic shift [21,24]. Concurrently, the field of immunometabolism has elucidated how immune cells, such as macrophages and T lymphocytes, drive and sustain pulmonary vascular inflammation and remodeling through their own metabolic reprogramming (e.g., a switch to glycolysis) [22,23]. A complex, bidirectional crosstalk exists between systemic metabolic dysregulation (e.g., insulin resistance, dyslipidemia) and immune inflammation, mediated by cytokines, extracellular vesicles, and other signaling molecules, creating a feed-forward loop that propels disease progression [2,8]. Therefore, PAH should be redefined as a syndrome of systemic metabolic–immune network dysfunction, with the pulmonary vascular manifestations representing merely the tip of the iceberg [6,139].

### 7.2. The Imperative for a Multimodal Treatment Paradigm

The current therapeutic paradigm, predominantly centered on targeting endothelial dysfunction with vasodilatory and anti-proliferative agents, while providing symptomatic relief, fails to reverse vascular remodeling or correct the underlying metabolic–immune disequilibrium, highlighting its inherent limitations [3]. Consequently, future treatment strategies must pivot from a purely “vasocentric” approach to an integrated model that combines systemic homeostasis restoration with localized vascular targeting. This multimodal paradigm includes the following: (1) Metabolic Homeostasis Restoration: Investigating the role of metabolic modulators such as metformin [140] and SGLT2 inhibitors [141] in improving systemic insulin sensitivity, mitochondrial function, and right ventricular energetics; (2) Immunometabolic Modulation: Targeting specific pathways like macrophage glycolysis [22], T-cell metabolic enzymes [23], or the complement system [124] to disrupt pro-inflammatory cycles; (3) Lifestyle and Microbiome Interventions: Implementing personalized exercise regimens [142], dietary modifications (e.g., Mediterranean diet) [139], and gut microbiota modulation [143] to systemically improve metabolic and immune status. Ultimately, these strategies must be synergistically combined with existing vascular-targeted therapies to form a multi-layered, individualized treatment framework capable of more effectively halting or reversing the disease process.

### 7.3. Final Outlook on the Evolving Field

PAH research stands at a transformative crossroads. Emerging technologies—including single-cell and spatial transcriptomics, multi-omics integration, and artificial intelligence—are uncovering the cell-specific metabolic and immune landscapes of PAH with unprecedented resolution, paving the way for novel biomarker and therapeutic target discovery [23,26]. The move towards personalized medicine is inevitable, requiring the integration of a patient’s genetic background (e.g., BMPR2 or SOX17 mutations) [2,144], specific metabolic profile [26], and immune phenotype [145,146] to enable “tailor-made” precision therapy. Significant challenges remain, however, including clarifying the causal relationship between metabolic dysregulation and PAH [145], overcoming barriers to clinical translation such as the standardization of diagnostic tools and drug accessibility [147,148], and ensuring ethical equity in research [4,149]. Future priorities must include well-designed longitudinal studies to establish causality, the development of combination therapies targeting multiple pathways, and the validation of novel strategies through large-scale clinical trials in diverse patient populations [3,150]. By deepening our understanding of the role of systemic metabolic and immune networks in PAH, we have the potential not only to revolutionize therapeutic approaches but also to fundamentally reshape the conceptual framework of this complex disease, ultimately delivering substantial improvements in patient quality of life and long-term outcomes.

## Figures and Tables

**Table 1:** Summary of Clinical Epidemiological Data on Systemic Metabolic Abnormalities in PAH Patients.

Metabolic Abnormality	Prevalence/Characteristics in PAH Patients	Association with Disease Severity or Prognosis
Insulin Resistance/Diabetes	Insulin resistance present in ~51.4% of patients [9,10]	Associated with severity of liver fibrosis, reduced exercise capacity; may independently predict outcomes [11,12,13]
Obesity (Obesity Paradox) ^1^	Some obese patients exhibit better survival [4,5]	Linked to adipose distribution (subcutaneous vs. visceral) and adipokine profile (adiponectin/leptin ratio) [14,15,16]
Muscle Wasting	Affects up to 60% of PAH patients [17,18]	Strongly associated with increased mortality [17]; a marker of disease severity
Liver Dysfunction/Cholestasis	42.3% had elevated GGT; 38.1% had elevated ALP [19]	Hyperbilirubinemia is an independent predictor of mortality (HR = 4.29) [19]
Dyslipidemia	Low HDL and high triglycerides are common [10,20]	Associated with worsening right ventricular function [20]

^1^ The “obesity paradox” refers to the observed phenomenon where, in certain PAH populations, obesity is associated with improved survival despite its known pro-inflammatory and pro-remodeling effects.

**Table 2:** Promising Metabolic and Immunometabolic Biomarkers in PAH.

Biomarker Category	Specific Biomarker	Change/Level in PAH	Clinical Significance/Association	Study Type	Sample Size	References
Adipokines	Adiponectin /Leptin Ratio	Decreased ratio driven by elevated leptin and reduced adiponectin. Importantly, this imbalance has been observed even in non-obese (lean) PAH patients, suggesting it is not merely a consequence of adiposity [16,101].	Reflects adipose tissue dysfunction; linked to vascular inflammation and remodeling; high leptin/adiponectin ratio correlates with disease severity [16,31].	Cross-sectional/Clinical Cohort	PAH: 50–150, Controls: 30–100	[16,31,101]
Inflammatory Cytokines	IL-6, TNF-α	Persistently elevated in circulation [7,8]	Markers of systemic inflammation; drive endothelial dysfunction, immune cell activation, and are associated with worse outcomes [7,8,72].	Clinical Cohort	PAH: 40–200	[7,8,72]
Metabolite	Lactate	2.7 ± 0.5 mmol/L (PAH) vs. 1.2 ± 0.3 mmol/L (Control) [118]	Promotes macrophage M2 polarization via MCT1; associated with exercise intolerance and disease severity [118].	Clinical Cohort/Case–Control	PAH: ~60, Controls: ~30	[118]
Tryptophan Metabolite	Kynurenine	2.8 μmol/L (PAH) vs. 1.9 μmol/L (Control) [99]	Modulates Th17/Treg balance; associated with immune dysregulation and may serve as a marker of IDO activity [99].	Cross-sectional	PAH: 41, Controls: 20	[99]
Complement System	Complement C3a	Elevated 2.8-fold (compared to controls) [124]	Associated with disease progression and pro-inflammatory remodeling; a potential prognostic marker [124].	Clinical Cohort	PAH: ~70, Controls: ~30	[124]
Muscle Atrophy-Related	GDF11	Serum levels elevated in PAH patients [56]	Induces muscle atrophy via STAT3 pathway; correlates with muscle wasting and poor outcomes [56].	Clinical Cohort/Preclinical	PAH: 30–50	[56]
Liver-Derived Factor	MIF (Macrophage Migration Inhibitory Factor)	Elevated in PoPH patients; correlates with PVR (r = 0.58) [43]	Promotes PASMC proliferation; involved in pulmonary vascular remodeling; potential biomarker for PoPH [43,44].	Clinical Cohort	PoPH: 27, iPAH: 18, Controls: 10	[43,44]

Values are presented as reported in the cited references; *p*-values for comparisons are provided in the main text.

**Table 3:** Therapeutic Strategies Targeting the Systemic Metabolic and Immunometabolic Axis in PAH.

Therapeutic Strategy Class	Representative Agent /Intervention	Primary Mechanism of Action	Potential/Status in PAH
Repurposed Antidiabetic Agents	SGLT2 Inhibitors (e.g., Empagliflozin)	improves insulin sensitivity; reduces weight; anti-inflammatory; improves endothelial function.	Strong preclinical and cardioprotective evidence; dedicated PAH trials underway
	GLP-1 Receptor Agonists (e.g., Liraglutide)	similar to SGLT2i; promotes GLP-1 secretion.	High potential; requires validation in PAH clinical trials
	Metformin	activates AMPK; inhibits mTOR; reverses “Warburg effect,”; improves mitochondrial function.	Effective in preclinical models; may be considered for PAH patients with insulin resistance
Direct Immunometabolic Modulators	PFKFB3 Inhibitors (e.g., PFK158)	Precisely inhibits a key glycolytic enzyme; dampening M1 macrophage inflammatory response;	Preclinical stage; represents next-generation metabolic intervention
	Pyruvate Kinase M2 (PKM2) Inhibitors	inhibits glycolysis; interferes with HIF-1α signaling; targets both vascular and immune cells.	Preclinical research stage
	DHODH Inhibitors	blocks de novo pyrimidine synthesis; inhibiting lymphocyte over-proliferation.	Preclinical research stage; potentially for immune-active subtypes
	Itaconate and its derivatives	activates NRF2; exerts antioxidant; anti-inflammatory effects; mimics endogenous feedback.	Novel “metabolo-immunomodulatory” strategy; preclinical research
Lifestyle Interventions	Personalized Exercise Rehabilitation	improves skeletal muscle; mitochondrial function; insulin sensitivity; releases anti-inflammatory myokines.	Foundation of comprehensive PAH management; strong evidence base
	Medical Nutrition Support (e.g., Mediterranean Diet)	improves overall metabolic health; anti-inflammatory; corrects specific deficiencies (e.g., iron).	Important adjunct, especially for catabolic state
Targeted Delivery & Emerging Frontiers	Ligand-conjugated Nanoparticles	enables drug delivery specific to pulmonary vasculature or right ventricle; improving efficacy/safety.	Future technological direction
	Microbiome Modulation (Probiotics/Prebiotics)	Modulates host immune metabolic homeostasis via metabolites like short-chain fatty acids.	Emerging frontier; preclinical evidence shows potential

“Preclinical stage” indicates evidence primarily from in vitro or animal models of PAH. “Emerging frontier” denotes concepts with promising preliminary data but not yet ready for clinical translation.

## Data Availability

No new data were created or analyzed in this study.

## Abbreviations

The following abbreviations are used in this manuscript:

2-DG	2-Deoxyglucose
3-BrPA	3-Bromopyruvate
16αOHE1	16α-Hydroxyestrone
AGEs	Advanced Glycation End-products
ALDH1A3	Aldehyde Dehydrogenase 1 Family Member A3
ALP	Alkaline Phosphatase
AMPK	AMP-activated Protein Kinase
BCAAs	Branched-Chain Amino Acids
BMPR2	Bone Morphogenetic Protein Receptor Type 2
cAMP	Cyclic Adenosine Monophosphate
CBD	Cannabidiol
CCR7	C-C Chemokine Receptor Type 7
CHD-PAH	Congenital Heart Disease-associated PAH
CTD-PAH	Connective Tissue Disease-associated PAH
CTEPH	Chronic Thromboembolic Pulmonary Hypertension
DHODH	Dihydroorotate Dehydrogenase
FAO	Fatty Acid Oxidation
FDG-PET	Fluorodeoxyglucose Positron Emission Tomography
FFAs	Free Fatty Acids
FoxO1	Forkhead box protein O1
FoxP3	Forkhead box P3
FXR	Farnesoid X Receptor
GDF11	Growth Differentiation Factor 11
GGT	γ-Glutamyltransferase
GLP-1 RAs	Glucagon-like Peptide-1 Receptor Agonists
HIF-1α	Hypoxia-Inducible Factor-1α
HMW	High-Molecular-Weight
IDO	Indoleamine 2,3-Dioxygenase
IL-1β	Interleukin-1β
IL-6	Interleukin-6
IL-10	Interleukin-10
IL-17	Interleukin-17
iPAH	Idiopathic Pulmonary Arterial Hypertension
iNOS	Inducible Nitric Oxide Synthase
JAK2	Janus Kinase 2
STAT3	Signal Transducer and Activator of Transcription 3
MAPK	Mitogen-Activated Protein Kinase
MCT1	Monocarboxylate Transporter 1
MIF	Macrophage Migration Inhibitory Factor
Mfn2	Mitofusin 2
mTOR	Mechanistic Target of Rapamycin
MuRF1	Muscle RING-finger protein-1
NAFLD	Non-Alcoholic Fatty Liver Disease
NETs	Neutrophil Extracellular Traps
NF-κB	Nuclear Factor kappa-light-chain-enhancer of activated B cells
NLR	Neutrophil-to-Lymphocyte Ratio
NO	Nitric Oxide
NT-proBNP	N-terminal pro-B-type Natriuretic Peptide
NYHA	New York Heart Association
ObR	Leptin Receptor
OXPHOS	Oxidative Phosphorylation
PAH	Pulmonary Arterial Hypertension
PASMCs	Pulmonary Arterial Smooth Muscle Cells
PBMCs	Peripheral Blood Mononuclear Cells
PCr	Phosphocreatine
PDK	Pyruvate Dehydrogenase Kinase
PFK	Phosphofructokinase
PFKFB3	6-Phosphofructo-2-kinase/fructose-2,6-bisphosphatase 3
PI3K/Akt	Phosphoinositide 3-kinase/Protein Kinase B
PKM2	Pyruvate Kinase M2
PoPH	Portopulmonary Hypertension
PPARα/γ	Peroxisome Proliferator-Activated Receptor alpha/gamma
PVR	Pulmonary Vascular Resistance
ROS	Reactive Oxygen Species
RV	Right Ventricle/Ventricular
SAT	Subcutaneous Adipose Tissue
SGLT2i	Sodium-Glucose Cotransporter-2 Inhibitors
SOX17	SRY-Box Transcription Factor 17
SSc-PAH	Systemic Sclerosis-associated PAH
TCA cycle	Tricarboxylic Acid Cycle
Tfh cells	T Follicular Helper cells
TGR5	Takeda G protein-coupled receptor 5
Th17 cells	T helper 17 cells
TNF-α	Tumor Necrosis Factor-alpha
Tregs	Regulatory T cells
VAT	Visceral Adipose Tissue
VEGF	Vascular Endothelial Growth Factor
VO_2max_	Maximal Oxygen Uptake
α-KG	Alpha-Ketoglutarate
GPR40/FFAR1	G protein-coupled receptor 40/Free Fatty Acid Receptor 1
